# Report of Five Years of Experience in Neonatal Screening for Mucopolysaccharidosis Type I and Review of the Literature

**DOI:** 10.3390/ijns6040085

**Published:** 2020-11-02

**Authors:** Vincenza Gragnaniello, Daniela Gueraldi, Laura Rubert, Francesca Manzoni, Chiara Cazzorla, Antonella Giuliani, Giulia Polo, Leonardo Salviati, Alberto Burlina

**Affiliations:** 1 Division of Inherited Metabolic Diseases, Department of Diagnostic Services, University Hospital of Padua, via Orus 2/B, 35129 Padua, Italy; vincenza.gragnaniello@aopd.veneto.it (V.G.); daniela.gueraldi@aopd.veneto.it (D.G.); laura.rubert@aopd.veneto.it (L.R.); francesca.manzoni@aopd.veneto.it (F.M.); chiara.cazzorla@aopd.veneto.it (C.C.); antonella.giuliani@aopd.veneto.it (A.G.); giulia.polo@aopd.veneto.it (G.P.); 2 Clinical Genetics Unit, Department of Diagnostic Services, University Hospital of Padua, via Giustiniani 2, 35129 Padua, Italy; leonardo.salviati@unipd.it

**Keywords:** mucopolysaccharidosis type I, expanded newborn screening, lysosomal disorders, second-tier test, tandem mass spectrometry, α-L-iduronidase, glycosaminoglycans, dermatan sulfate, heparan sulfate

## Abstract

Mucopolysaccharidosis type I (MPS I) is a progressive lysosomal storage disease, with neurological and visceral involvement, in which early diagnosis through newborn screening (NBS) and early treatment can improve outcomes. We present our first 5 years of experience with laboratory and clinical management of NBS for MPS I. Since 2015, we have screened 160,011 newborns by measuring α-L-iduronidase (IDUA) activity and, since 2019, glycosaminoglycans (GAGs) in dried blood spot (DBS) as a second-tier test. Positive screening patients were referred to our clinic for confirmatory clinical and molecular testing. We found two patients affected by MPS I (incidence of 1:80,005). Before the introduction of second-tier testing, we found a high rate of false-positives due to pseudodeficiency. With GAG analysis in DBS as a second-tier test, no false-positive newborns were referred to our clinic. The confirmed patients were early treated with enzyme replacement therapy and bone-marrow transplantation. For both, the clinical outcome of the disease is in the normal range. Our experience confirms that NBS for MPS I is feasible and effective, along with the need to include GAG assay as a second-tier test. Follow-up of the two positive cases identified confirms the importance of early diagnosis through NBS and early treatment to improve the outcome of these patients.

## 1. Introduction

Mucopolysaccharidosis type I (MPS I) is an autosomal-recessive lysosomal storage disease (LSD) caused by a deficiency of the enzyme α-L-iduronidase (IDUA), involved in the degradation of two glycosaminoglycans (GAGs), dermatan (DS) and heparan sulfate (HS) [1]. The deficiency leads to progressive neurocognitive decline, short stature with bone deformities, hepatosplenomegaly, cardiomyopathy and shortened life span [1,2,3,4]. Diagnosis is based on a pattern of urinary GAGs (elevated HS and DS levels), enzyme deficiency (in dried blood spot (DBS), leukocytes or lymphocytes) and genetic analysis [3]. Available treatments include enzyme replacement therapy (ERT) with laronidase, allogenic hematopoietic stem cell transplantation (HSCT) and ex vivo gene therapy in autologous hematopoietic stem cells. HSCT before age 1 to 2 years is the recommended treatment to prevent cognitive impairment in patients with the severe form of MPS I [5,6,7]. These treatments can significantly improve the outcomes in patients with MPS I, but, due to the progressive nature of this disease, an early diagnosis can change the natural history of the disease. Based on these characteristics and the estimated incidence of approximately 1 in 100,000 live births [8], newborn screening (NBS) for MPS I has been proposed since 2010. Two approaches to determine IDUA activity in DBS have been used. The first method was a fluorometric assay that identifies samples with reduced enzyme activity using a fluorescently labelled artificial substrate (4-methylumbelliferyl 4-MU) [7,9]. A limitation of this method is that it has restricted capacity for multiplex testing to assay multiple lysosomal enzymes simultaneously, although this problem was partially overcome by the development of digital microfluidic chip technology [10]. Tandem mass spectrometry (MS/MS) technology has been proposed, because it allows the simultaneous quantification of several enzyme activities in a single DBS. This procedure reduces times and costs and allows rapid screening of large populations. The most important MPS I pilot studies and screening programs worldwide are summarized in Table 1. 

Some of them did not identify affected newborns. The first pilot program was conducted in Taiwan from October 2008 to April 2013, using a fluorometric assay. Of 35,285 screened newborns, two positive patients were found (incidence 1:17,643) [9]. In Missouri, a fluorometric assay was implemented in 2013 to screen for MPS I using a multiplex technology (digital microfluidic method) that assesses three other lysosomal storage diseases (LSDs). Two affected newborns were identified among 308,000 screened (incidence 1:154,000). Two patients with genotypes of uncertain significance were also identified [13]. Subsequently, several programs using multiplex MS/MS technology were established. In Illinois between 2014 and 2016, one patient was diagnosed with MPS I out of 219,973 screened newborns [16]. Other studies involving a smaller number of newborns were conducted in North Carolina (estimated incidence 1:62,734), Kentucky (1:55,161) and Washington (1:35,700) [8,14,21]. This last study was a retrospective investigation using deidentified DBS. Of note, a second program in Taiwan conducted from 2015 to 2017 for MPS I and MPS II identified four patients with MPS I patients out of 294,196 screened newborns (incidence 1:73,543), with a false-positive rate of 0.037%. An enzyme assay for IDUA and iduronate 2-sulfatase was used [14]. Interestingly, another program in Japan used a GAG assay as a first-tier test and, due to an elevated false-positive rate (0.03%), employed an enzyme activity assay as a second-tier test to screen positive samples for MPS I and II. They did not find a false-positive for MPS I after adding the second-tier test, but this may be due to the small number of newborns screened [24]. The MPS I incidence in these studies ranged from 1/17,643 in Taiwan to 1/219,973 in Illinois, also based on geographical differences. Recently in the USA, MPS I and Pompe disease have been included in the Recommended Uniform Screening Panel (RUSP) for metabolic diseases. In Italy, several different studies have been conducted in the last ten years, revealing a high incidence of pseudodeficiency but identifying no patients [11,20]. We reported previously on our first 2 years of experience with MPS I newborn screening, which identified one patient among 44,411 screened newborn [18]. This paper describes our results after 5 years and over 160,000 newborns screened for MPS I, follow-up data on diagnosed patients, and discuss the role of second-tier testing. 

## 2. Materials and Methods

### 2.1. Screening Population

**Figure 1:**
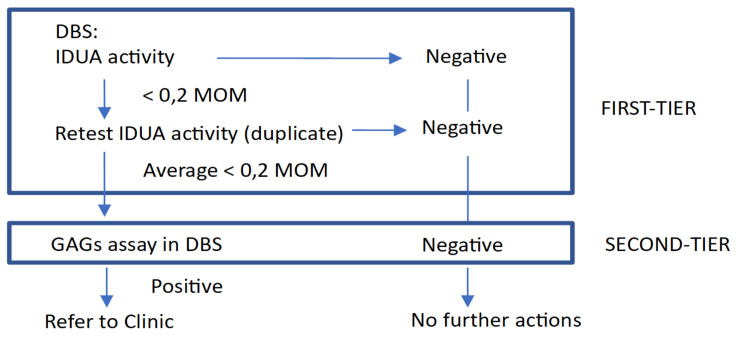
Two-tier Newborn Screening Algorithm for MPS I. DBS: dried bloot spot, IDUA: α-L-iduronidase, MOM: multiple of median, GAGs: glycosaminoglycans (dermatan sulfate, heparan sulfate).

In total, 160,011 consecutive newborn DBS samples were collected between September 2015 and September 2020 at the Regional Center for Expanded Newborn Screening, University Hospital of Padua. The study was reviewed by the Ethical Committee Board from University Hospital Padova and deemed exempt, as it was not considered research. Following our NBS protocol, samples were collected between 48 h–72 h of life on the same card on which the other NBS tests were performed and shipped 6 days per week at room temperature to our laboratory. DBS were assayed for enzyme deficiencies associated with MPS I, as well as Fabry disease, Gaucher disease, Pompe disease using the NeoLSDTM kit (PerkinElmer, Turku, Finland) as previously described [18]. We chose cut-offs of 0.2 multiples of median (MOM) on the basis of a pre-pilot study (from more than 3500 de-identified DBS samples) and in the first 9 months of screening. These cut-off values were chosen because they enabled the detection of positive cases while avoiding too many recalls from the newborn screening process. In 2019, GAGs quantification in DBS was a second-tier test for samples with low IDUA activity. GAGs levels (HS and DS) were measured by LC-MS/MS after methanolysis, as previously described [26]. Patients with low IDUA activity and elevated GAGs are referred to our unit for clinical and biochemical evaluation including genetic analysis (Figure 1).

### 2.2. Clinical and Biochemical Assessment

Newborns with positive MPS I screening results underwent confirmatory testing that included clinical examination, IDUA activity in lymphocytes, urinary GAGs and genetic analysis. DNA sequencing was carried out by next-generation sequencing (Illumia MiSeq, kit Agilent Haloplex), according to the manufacturer’s instructions, and identified variants were further verified by Sanger sequencing. Confirmed patients were evaluated with a neurological and psychomotor assessment, abdominal ultrasound, electrocardiogram, echocardiography, full-body X-ray for bone deformities, brain and spine MRN, audiometry and ophthalmologic assessment. Treatment decisions regarding ERT and/or HSCT were based on these results, considering disease severity. Periodic follow-up with clinical, biochemical and instrumental evaluations is shown in Table 2. 

Family members were counselled and offered testing and medical assessments, in particular, IDUA molecular testing to determine carrier status was performed on both parents. 

## 3. Results

### 3.1. MPS I Screening Results

Of the 160,011 newborns screened for MPS I, 27 (0.017%) had low enzyme activity in DBS (range 0.1 to 2.21 µM/h) at mean age of 5.57 ± 1.95 days (Table 3).

Normal values: IDUA > 2.3 µM/h (recalculated monthly due to slight seasonal variations); Urinary GAGs DS < 38.1 mg/mmol creatinine, HS < 4.6 mg/mmol creatinine; DBS GAGs DS < 2.7 mg/L, HS < 3.2 mg/L; NA not available, NP not performed, VUS variant of uncertain significance. 

MPS I was confirmed in two newborns by measuring IDUA activity in lymphocytes and GAGs in DBS and urine, as well as genetic analysis (patients 2 and 4). Both patients had very low enzyme activity in DBS (1.6% and 2.1% of the normal mean). This was confirmed by high excretion of GAGs in urine (HS 148.9 mg/mMol creatinine; DS 172.0 mg/mMol creatinine and HS 121.9 mg/mMol creatinine; DS 80.4 mg/mMol creatinine respectively, nv HS < 4.6mg/mMol creatinine; DS < 38.1 mg/mMol creatinine); both patients had known pathogenic mutations in both alleles. We found an overall MPS I incidence of 1 in 80,005 births. During the first 4 years of screening, when the only test was IDUA activity in DBS, the recall rate was 0.055%, with a false-positive rate of 0.053%. The positive predictive value (PPV) was 7.4%. This was due to the high incidence of pseudodeficient alleles. In the last 18 months, we employed a second-tier test. When IDUA activity was below the cut-off (2.3 µM/L), GAG analysis in DBS was performed. The recall rate during this period was 0.014% and no false-positives were referred to the clinic. We also retrospectively analysed GAGs in DBS from 22 previously recalled neonates with low IDUA screening values and found elevated GAGs only in two neonates with confirmed MPS I, but none of the patients with pseudodeficiency; the predictive value was 100%. Of the 27 recalled newborns, two were confirmed with MPS I (cases 2 and 4), two had mutations of unknown clinical significance (cases 5 and 12), while the other neonates had pseudodeficient alleles (two of them, cases 7 and 16, were compound heterozygotes with one pseudodeficient allele and one pathogenic allele, case 27 carried only one pseudodeficient allele). Except for the two affected patients, all others had normal clinical examinations and urinary GAGs. The most common pseudodeficient allele was p.A79T (present in 16/17 cases, 21/34 alleles). Interesting, all newborns with pseudodeficiency were of African origin, except for case 16, which was a compound heterozygote with the p.H82Q pseudodeficiency variant and a known pathogenic mutation. IDUA enzyme activity on DBS of these patients ranged from 0.1 to 2.21 mMol/hour, overlapping that of patients with MPS I. For newborns with pseudodeficiency, no further follow-up was requested. None of the infants with variance of uncertain significance (VUS) had early-onset MPS I. For these patients, we established a 12-month follow-up appointment, although some parents refused to continue follow-up, as children were normal; we thus informed paediatricians to be alert for abnormal clinical symptoms suggestive of the disease.

### 3.2. Clinical Follow-Up

Two patients showed biochemical and genetic characteristics of MPS I Hurler form and Hurler/Scheie form:

-Patient A, a female of African origin (Morocco), is now 3.5 years old. She was asymptomatic on initial evaluation at 15 days of age, but at confirmatory testing, she had elevated urinary GAGs (HS 148.9 mg/mMol creatinine, nv < 4.6; DS 172.0 mg/mMol creatinine, nv < 38.1) and genetic testing revealed that she was homozygous for the p.P533R mutation common in North African patients and previously reported to be associated with the Hurler/Scheie phenotype [26]. The mutation was confirmed in both parents, who were consanguineous. The results of further assessments are summarized in Table 4A. The patient had diffuse corneal clouding at birth, oval-shaped lumbar vertebral bodies, mild mitral insufficiency at echocardiogram and mild bilateral conductive hearing loss. In the first months of life, she suffered from recurrent respiratory infections that required hospitalization and oxygen therapy. She began ERT with laronidase (100 U/kg weekly) one month after birth and responded very well to treatment. Her urinary GAGs had almost normalized after 1 month of therapy (HS 9.2 mg/mMol creatinine, nv < 4.6; DS 15.4 mg/mMol creatinine, nv < 38.1). We continued her follow up, with clinical and biochemical evaluation every 3 months for the first year and subsequently every 6 months, with periodical instrumental assessments. Currently, she is asymptomatic, except for the persistence of diffuse corneal clouding, which is known to be stabilized but not reversed by ERT [27]. Her hearing normalized and she has normal growth and psychomotor development. Full body X-ray, abdominal ultrasound and brain MRI are normal. Cardiac assessment with electrocardiogram and echocardiogram does not show abnormalities. Urinary GAGs pattern has been consistently normal.

-Patient B, a female of Italian origin, is now 2.5 years old. Her characteristics are described in Table 4B. On initial examination at 15 days of life, she had clinical features of a severe form of disease: corneal clouding, coarse facial features, moderate sensorineural hearing loss, poor growth, rounding of the iliac wings and large cisterna magna at brain MRI. This phenotype was confirmed by elevated urinary GAGs (HS 121.9 mg/mMol creatinine, nv < 4.6; DS 80.0 mg/mMol creatinine, nv < 38.1); genetic analysis of the IDUA gene revealed compound heterozygosity for two known pathogenic mutations (c.46–57del12/p.Y201X) consistent with Hurler syndrome. The carrier status of both parents was confirmed. ERT with laronidase (100 U/kg weekly) was started at 1 month of life, with a good response. After 3 months of therapy, urinary GAGs had almost normalized (HS 21.7 mg/mMol creatinine, nv < 27.9; DS 29.2 mg/mMol creatinine, nv < 14.7). Due to the severe phenotype, at 6 months of age, she received an allogenic HSCT. She lacked a matched sibling donor, thus she received a fully matched identical unrelated cord blood (UCB) transplantation (12/12, high resolution), according to donor hierarchy, after conditioning with antithymocyte globulin, fludarabine and busulfan. She received 1.17 × 10^8^ /kg total nucleated cells and 6.3 × 10^5^/kg CD34+ cells. The procedure was performed without complications and, after one month, the patient achieved high donor chimerism (92% donor, 8% recipient) and normal IDUA levels (3.72 µM/h), that persist after 2 years (chimerism 97% donor, 3% recipient, enzyme activity 6.70 µM/h). GAGs remained normal after suspension of ERT, 6 months after HSCT. Currently, she has no neurologic involvement and normal psychomotor development. Her brain and spine MRIs are normal, except for mild thickening of the tissues around the odontoid process that does not restrict the spinal canal. Growth has improved and hearing normalized. Abdominal ultrasound shows no enlargement of liver or spleen, and cardiac assessment with electrocardiogram and echocardiogram is normal for her age. Although GAG excretion is normal, she has mild coarse facial features, a small umbilical hernia, some skeletal abnormalities (rounded thoracic and lumbar vertebral bodies) and corneal clouding.

## 4. Discussion

Newborn screening for LSDs has gained increasing importance as the number of therapeutic options has expanded and the evidence base supporting improved outcomes with early intervention has been clearly demonstrated for MPS I [28,29].

In North-East Italy, screening for MPS I, together with Pompe, Fabry and Gaucher, using the multiplexed NeoLSDTM assay system (PerkinElmer) has been a feasible and effective element of our expanded neonatal screening program since 2015. In our previous study, the incidence of MPS I was 1 in 44,411 neonates. That study identified 12 false-positive newborns (1:3700). This was due to the high incidence of pseudodeficiency [18], characterized by low enzyme activity, but normal urinary GAGs, no clinical findings and molecular mutations characterized by a high allele frequency in the normal population [8,16,20]. This unexpectedly high number of false-positive for MPS I due to pseudodeficiency prompted us to develop a second-tier test to quantify GAG levels in dried blood spots [26]. In the present study, we report our first five years of experience with NBS for MPS I, initially by measuring IDUA enzyme activity in DBS specimens and for the last 18 months with the second-tier test for GAGs in DBS. The combined approach demonstrates its potential to reduce the screen-positive samples if utilized prospectively by eliminating patients with only pseudodeficient variants. Of the newborns, 2/27 with low IDUA activity were confirmed to have MPS I with pathogenic variants consisting of a severe form of disease (Hurler syndrome, patient 2) and an intermediate form (Hurler/Sheie syndrome, patient 4). Of the neonates, 25/27 with positive results on initial screening subsequently underwent full diagnostic assessment including determination of IDUA activity in lymphocytes, urinary GAGs by LC-MS/MS analysis and genotyping. All patients with genotypes that were consistent with pseudodeficiency (*n* = 21), compound heterozygotes (*n* = 2), and variants with unknown significance (*n* = 2) had normal results for GAGs in DBS. Pseudodeficient alleles for MPS I are more prevalent in African and African-American populations (most common p.A79T, with an allele frequency about 4% for the African population) [20,29,30] and this was confirmed in our study, in which all except one case were of African ethnicity and carried at least one p.A79T allele (homozygous in five cases). Only one newborn was Italian (case 16), and he carried the p.H82Q mutation, which has an allele frequency of about 0.5% in European populations (Exome Aggregation Consortium database). The large population with African ethnicity in Italy explains the high incidence of pseudodeficiency that we found in our population (0.8% of positive newborns), which is more than originally estimated. The high number of screen-positive specimens necessitated modification of the screening algorithm. Because the range of residual enzyme activities in true-positive and pseudodeficient cases overlap almost completely, this marker alone is not suited for differential diagnosis. Several strategies can be used to reduce recall rates in MPS I screening, such as the use of post-analytical tools or second-tier tests, including GAGs and genetic testing [8,21]. Recently, Taylor et coll. showed that sequencing of the IDUA gene from a second DBS punch can be used as a second-tier test [8]. It reduced the screen-positive samples by eliminating patients with only pseudodeficiency, but can be inconclusive when variants of unknown significance are encountered. Moreover, genetic analysis is more expensive and requires more time than biochemical assays. Since February 2019, we have used GAGs quantification on DBS as our second-tier test. Accumulation of GAGs starts before birth (GAGs can be found in chorionic villi, the foetus and amniotic fluid) and their levels tend to be highest from 0 to 6 months of age [31,32] so that their quantification can be used in NBS programs [26]. Our two-test algorithm with the sequential determination of enzyme activity and GAGs reduced false-positives and recall rates compared to the first years of our program (0.014% vs. 0.055%). Our second-tier test was able to discriminate between MPS I and pseudodeficiency in retrospective testing of patients that had been recalled for low IDUA activity with a negative predictive value of 100%.

The follow-up of our two patients confirms the importance of early diagnosis and treatment. Allogenic HSCT is the only treatment proven to prevent progressive neurodegenerative disease in Hurler patients [33]. Our patient B shows that early transplantation can change the natural history of the disease. While this procedure has been shown to prevent irreversible damage due to MPS, it is also associated with a small risk of complications. Younger age at transplantation is associated with a reduction of complications [34], further justifying NBS.

Patient B was treated with ERT and received a UCB transplantation at 6 months of age without complications. She had no siblings, so we chose a UCB donor graft because they are readily accessible, reducing the time for donor searching. Additionally, several studies indicate that UCB transplantation leads to higher full donor chimerism rates, thereby predicting better enzyme levels in the long-term compared with bone marrow and peripheral blood stem cells [33,34]. Our patient confirms this. She achieved high chimerism and IDUA activity that is maintained 2 years after the transplantation. At 2.5 years of age, she has no neurologic involvement or psychomotor delay and has only mild features of MPS I, in particular bone deformities. Long-term data on patients treated early with HSCT do not support or exclude the need for ERT to treat bone lesions [35].

Patient A has an intermediate form of the disease. Although recent evidence suggests the possibility of HSCT also in patients with Hurler/Scheie form [34], this patient had no matched family donors and responded very well to ERT. After 3.5 years, she only suffers from corneal clouding, already present at birth and known to be non-responsive to ERT [27]. No new symptoms have developed. The importance of early diagnosis and treatment has been demonstrated by studies on siblings. Gabrielli et al. reported a better outcome after a 12-year follow-up in a patient who initiated ERT treatment at 5 months of age compared to a sibling who had started ERT at 5 years of age [36]. Similar conclusions were made by Laraway et al., who reported on three siblings with MPS I, of which the younger, treated with ERT from 4 months of age, had a better outcome [37], and by Al-Sannaa et al., who retrospectively analysed nine sibships affected by MPS I: older siblings were treated with ERT after the development of significant clinical symptoms (median age 7.9 years), while younger siblings, treated before significant symptomatology (median age 1.9 years), had a more notable improvement or stabilization of somatic signs and symptoms [38]. Recently, Yamazaki et al. described a younger sibling of a deceased Hurler patient. The younger sibling was diagnosed at 18 days of age, started ERT at 34 days, and underwent HSCT at 9 months of age. After 5 years, she had only mild signs of dysostosis multiplex and mild cardiac valvular disease [39]. This patient had been identified because of an older affected sibling; however, NBS allows early diagnosis and treatment for the entire population, not only siblings of affected patients.

Our study has several limitations. The number of neonates tested is still limited for defining the incidence of a rare disease, although our results are similar to the frequency of MPS I in the Italian population reported by Dionisi Vici et al. [40]. The correct management of patients with VUS is still unclear. Moreover, some patients with attenuated forms may have low urinary GAG levels [8] and sufficient data on levels at birth in these patients are lacking [41], so we cannot exclude that milder forms may be missed. Such patients may not be ascertained until they become symptomatic and are diagnosed years later. However, Rujter et al. reported two patients with attenuated MPS I and elevated GAGs in newborn DBS [42]. Moreover, the lack of identification of late-onset phenotypes may not be a disadvantage. In effect, neonatal diagnosis of late-onset LSDs presents an ethical dilemma, because decades can pass before the onset of symptoms, resulting in anxiety for patients and parents, and unnecessary medical interventions. Medical management is complicated by clinical heterogeneity, the inability to predict phenotype and the lack of consensus about when to begin treatment in mild forms [20,30].

In conclusion, our 5-year experience confirms the importance of including MPS I in NBS programs. Our protocol using an MS/MS assay system is feasible, effective and easily integrated into the workflow of laboratories using MS/MS. Our algorithm including IDUA activity and a GAGs assay on DBS as a second-tier test decreased the false-positive rate in newborns referred to our Clinic. Our experience also outlines the importance of early diagnosis in improving disease outcomes when specific treatments, such as ERT and/or HSCT, are available. Authors should discuss the results and how they can be interpreted from the perspective of previous studies and of the working hypotheses. The findings and their implications should be discussed in the broadest context possible. Future research directions may also be highlighted.

## Figures and Tables

**Table 1:** Worldwide newborn screening for Mucopolysaccharidosis type I (MPS I).

Years	Region	Methodology	Screened Disorders Other Than MPS I	Screened Newborns	False-Positive Rate	Incidence
2008–2013 [9]	Taiwan	Fluorometric enzymatic assay	/	35,285	0.048%	1/17,643
2010–2012 [11]	Italy	Fluorometric enzymatic assay	Pompe disease, Fabry disease, Gaucher disease	3403	0.088%	/
2012–2016 [12]	Mexico ^1^	MS/MS enzymatic assay	Pompe disease, Fabry disease, Gaucher disease, Niemann Pick type A/B, Krabbe disease	20,018	0.009%	/
Since 2013 [13]	Missouri	Digital microfluidic fluorometric enzymatic assay	Pompe disease, Fabry disease, Gaucher disease	308,000	0.040%	1/154,000
2013 [14]	Washington ^2^	MS/MS enzymatic assay	Fabry disease, Pompe disease	106,526	0.006%	1/35,700
2015–2016 [15]	New York	MS/MS enzymatic assay	Pompe disease, Fabry disease, Gaucher disease, Niemann Pick type A/B	35,816	0.036%	/
2014-2016 [16]	Illinois	MS/MS enzymatic assay	Pompe disease, Fabry disease, Gaucher disease, Niemann Pick type A/B	219,973	0.068%	1/219,973
2016 [17]	Washington ^2^	MS/MS enzymatic assay	Pompe disease, Fabry disease, Gaucher disease, Niemann Pick type A/B, Krabbe disease	About 43,000	0.014%	/
2015–2017 [18]	Italy	MS/MS enzymatic assay	Pompe disease, Fabry disease, Gaucher disease	44,411	0.027%	1/44,411
2015–2017 [19]	Taiwan	MS/MS enzymatic assay	MPS II	294,196	0.004%	1/73,549
2015–2017 [20]	Italy	MS/MS enzymatic assay	Pompe disease, Fabry disease	64,907	0.012%	/
2016–2017 [8]	North Carolina	MS/MS enzymatic assay	/	62,734	0.028%	1/62,734
2016 [21]	Kentucky	MS/MS enzymatic assay	Pompe disease, Krabbe disease	55,161	0.002%	1/55,161
2017 [22]	Brazil ^3^	Digital microfluidic fluorometric enzymatic assay	Pompe disease, Fabry disease, Gaucher disease	10,567	0.019%	/
2017 [23]	Georgia	MS/MS enzymatic assay	Pompe disease	59,332	0.018%	/
2017 [24]	Japan	GAGs on DBS by MS/MS assay	MPS II	18,222	/	/
2018 [25]	Brazil^2^	Fluorometric enzymatic assay	MPS VI, Pompe disease, Fabry disease, Gaucher disease	834	1.3%	/

^1^ Petroleos Mexicanos Health Services; ^2^ deidentified dry blood spots (DBSs); ^3^ performed by a private laboratory.

**Table 2:** Clinical follow-up of patients with confirmed MPS I.

	Every 3 Months ^1^	Every 6 Months ^1^	Annually ^1^
Medical history	X		
Physical examination, including neurological assessment	X		
Intellectual function (IQ)			X
Routine biochemical tests	X		
Urinary GAGs	X		
Enzyme activity ^2^ and chimerism	Only after HSCT (2/month during the first 6 months, 1/month during the following year)		
Anti-ERT antibodies	X		
Abdominal ultrasound		X	
Cardiac assessment, including electrocardiogram and echocardiogram		X	
Audiometry		X	
Ophthalmologic assessment		X	
Full-body X-ray			X
Brain and spine MRN			X

^1^ these frequencies refer to the most severe patients in the first years of life, to monitor therapy response and disease progression. However, the follow-up program should be individualized. ^2^ if patients are also receiving ERT, samples should be collected before infusions.

**Table 3:** Biochemical, molecular analysis and follow up of infants with positive NBS for MPS I.

Patient No.	Year of Birth	Sex	Ethnic Origin	IDUA Activity I DBS (µM/h) ^1^	IDUA Activity II DBS (µM/h) ^1^	DBS GAGs DS/HS mg/L	Genotype	Phenotype	Urinary GAGs DS/HS (mg/mmol Creatinine)	Treatment	Outcome
1	2016	M	West Africa	0.10	0.13	1.52/1.13	p.A79T/p.A79T (pseudoMPS I)	Not affected	NP	No	Dismissed
2	2017	F	European	0.17	0.34	8.84/10.42	p.S16_A19del/p.Y201X	MPS I Hurler	80.4/121.9	ERT+HSCT	Progressive improvement, very mild features of MPS I
3	2018	M	South Asia	0.20	0.23	2.86/2.35	NP ^2^	Not affected	37.9/4.1	No	Failed to follow up
4	2017	F	North Africa	0.22	0.20	7.38/4.88	p.P533R/p.P533R	MPS I H/S	172.0/148.9	ERT	Progressive improvement, asymptomatic, except for corneal clouding
5	2017	F	North Africa	0.38	0.73	2.85/3.1	p.R628G/p.R628G (VUS)	Uncertain significance	6.8/2.0	No	12-month follow-up appointment (refused)
6	2018	M	West Africa	0.40	0.67	1.87/2.03	p.A79T/p.D223N (pseudoMPS I)	Not affected	29.1/3.4	No	Dismissed
7	2016	M	North Africa	0.41	0.62	1.54/1.05	p.A79T+p.A361T/p.Y581X (carrier- Pseudo MPS I)	Not affected	14.4/2.1	No	Dismissed
8	2019 (January)	F	West Africa	0.47	0.53	NA	NP ^2^	Not affected	27.6/3.6	No	Dismissed
9	2018	F	West Africa	0.49	0.47	2.02/1.91	p.A79T/p.A79T (Pseudo MPS I)	Not affected	19.6/3.4	No	Dismissed
10	2017	F	West Africa	0.53	0.57	1.75/2.03	p.A79T/p.D223N (Pseudo MPS I)	Not affected	NP	No	Dismissed
11	2016	M	West Africa	0.54	0.61	1.52/1.52	p.A79T+p.T99I/p.D223N (Pseudo MPS I)	Not affected	NP	No	Dismissed
12	2017	F	European	0.55	0.56	1.62/1.72	p.L526P/p.L526P (VUS)	Uncertain significance	9.1/1.8	No	12-month follow-up appointment (refused)
13	2016	F	West Africa	0.58	0.56	2.06/1.63	p.A79T/p.A361T (Pseudo MPS I)	Not affected	NP	No	Dismissed
14	2017	F	North Africa	0.59	1.02	1.56/1.25	p.R263W/p.P650L (Pseudo MPS I)	Not affected	19.8/1.4	No	Dismissed
15	2017	F	West Africa	0.66	0.70	1.75/1.53	p.A79T/p.A79T (Pseudo MPS I)	Not affected	35.0/5.3	No	Dismissed
16	2016	F	European	0.71	0.55	NA	p.S16_A19del/p.H82Q (Carrier- Pseudo MPS I)	Not affected	NP	No	Dismissed
17	2016	M	West Africa	0.72	0.84	1.71/1.58	p.A79T/p.A79T (Pseudo MPS I)	Not affected	NP	No	Dismissed
18	2018	M	North Africa	0.85	0.98	2.09/2.06	p.A79T/p.R263W (Pseudo MPS I)	Not affected	13.8/1.7	No	Dismissed
19	2018	M	NA	0.88	0.79	NA	NP ^2^	Not affected	21.0/1.8	No	Dismissed
20	2018	F	West Africa	0.92	1.01	2.01/1.77	p.A79T/p.V322E (Pseudo MPS I)	Not affected	16.8/0.8	No	Dismissed
21	2017	M	West Africa	0.97	1.51	1.86/1.48	p.A79T/p.F501L (Pseudo MPS I)	Not affected	9.9/1.7	No	Dismissed
22	2018	M	NA	1.07	1.21	1.6/1.67	p.A79T/p.R263W (Pseudo MPS I)	Not affected	23.2/2.5	No	Dismissed
23	2018	M	West Africa	1.1	1.36	1.93/1.55	p.A79T/p.S586F (Pseudo MPS I)	Not affected	13.6/1.3	No	Dismissed
24	2017	F	West Africa	1.14	1.39	2.75/2.37	p.A79T/p.A79T (Pseudo MPS I)	Not affected	13.7/1.6	No	Dismissed
25	2015	M	Nord Africa	1.16	1.19	2.07/1.1	P.R263W/p.S586F (Pseudo MPS I)	Not affected	NP	No	Dismissed
26	2018	M	European	1.26	1.56	1.89/1.53	NP ^2^	Not affected	19.4/1.6	No	Dismissed
27	2017	F	West Africa	2.21	2.30	1.46/1.67	p.A79T/wt (Pseudo MPS I)	Not affected	NP	No	Dismissed

^1^ values are the mean for duplicate runs of different punches from the same DBS. ^2^ Parents refused consent to perform the genetic analysis.

**Table 4:** **A.** Patient A follow-up; **B**. Patient B follow-up.

**A. Patient A Follow-Up**		
	**Pre-ERT**	**Last Follow-Up**
Physical examination, including neurological assessment	Recurrent respiratory infections in the first year, no neurological signs	Regular growth, no neurological signs
Intellectual function (IQ)–Bayley scale–III Ed.	Not performed	Developmental Quotient 100
Routine biochemical tests	Normal	Normal
Urinary GAGs	HS 148.9 mg/mMol creatinine (nv < 4.6) DS 172.0 mg/mMol creatinine (nv < 38.1)	HS 1.8 mg/mMol creatinine (nv < 1.2) DS 6.7 mg/mMol creatinine (nv < 11.4)
Abdominal ultrasound	Normal	Normal
Cardiac assessment: -Electrocardiogram -Echocardiogram	Normal Patent foramen ovale (normal for age) and mild mitral insufficiency	Normal Normal
Audiometry/auditory brainstem response	Mild bilateral conductive hearing loss	Normal
Ophthalmological assessment	Diffuse corneal clouding	Diffuse corneal clouding, strabismus
Full-body X-Ray	Oval shaped lumbar vertebral bodies	Normal
Brain and spine MRI	Normal	Normal
**B. Patient B Follow-Up**		
	**Pre-HSCT**	**Last Follow-Up**
Physical examination, including neurological assessment	Coarse facial features, poor growth	Coarse facial features, small umbilical ernia, normal growth, subtle flexion contractures of distal interphalangeal joints (reducible)
Intellectual function (IQ)–Bayley scale–III Ed.	Developmental Quotient 65	Developmental Quotient 90
Routine biochemical tests	Normal	Normal
Urinary GAGs	Initial values: HS 121.9 mg/mMol creatinine (nv < 4.6) DS 80.4 mg/mMol creatinine (nv < 38.1) Post-ERT, pre-HSCT: HS 21.7 mg/mMol creatinine (nv < 27.9) DS 29.2 mg/mMol creatinine (nv < 14.7)	HS 4.2 mg/mMol creatinine (nv < 1.2) DS 11.9 mg/mMol creatinine (nv < 11.4)
Enzyme activity	0.17 uM/h (nv 1.9–15)	6.70 uM/h (nv 1.9–15), max value 18 uM/h
Chimerism		3% recipient, 97% donor
Abdominal ultrasound	Normal	Normal
Cardiac assessment: -Electrocardiogram -Echocardiogram	Normal Patent foramen ovale (normal for age)	Normal Normal
Audiometry/auditorybrainstem response	Mild-moderate bilateral sensorineural and conductive hearing loss	Normal
Ophthalmological assessment	Diffuse corneal clouding	Diffuse corneal clouding
Full body X-Ray	Rounding of the iliac wings	Rounded thoracic and lumbar vertebral bodies
Brain and spine MRI	Large cisterna magna	Mild thickening of the tissues around the odontoid process, normal spinal canal
